# The multiple sclerosis visual pathway cohort: understanding neurodegeneration in MS

**DOI:** 10.1186/1756-0500-7-910

**Published:** 2014-12-15

**Authors:** Elena H Martínez-Lapiscina, Elena Fraga-Pumar, Iñigo Gabilondo, Eloy Martínez-Heras, Ruben Torres-Torres, Santiago Ortiz-Pérez, Sara Llufriu, Ana Tercero, Magi Andorra, Marc Figueras Roca, Erika Lampert, Irati Zubizarreta, Albert Saiz, Bernardo Sanchez-Dalmau, Pablo Villoslada

**Affiliations:** Center of Neuroimmunology, Institut d’Investigacions Biomèdiques August Pi i Sunyer (IDIBAPS) - Hospital Clinic of Barcelona, Casanova 145, Planta 3A, 08036 Barcelona, Spain; Department of Ophthalmology, Institut d’Investigacions Biomèdiques August Pi i Sunyer (IDIBAPS) - Hospital Clinic of Barcelona, Barcelona, Spain; Department of Neurology, Institut d’Investigacions Biomèdiques August Pi i Sunyer (IDIBAPS) - Hospital Clinic of Barcelona, Barcelona, Spain

**Keywords:** Multiple Sclerosis, Visual pathway, Neurodegeneration, Cohort studies

## Abstract

**Background:**

Multiple Sclerosis (MS) is an immune-mediated disease of the Central Nervous System with two major underlying etiopathogenic processes: inflammation and neurodegeneration. The latter determines the prognosis of this disease. MS is the main cause of non-traumatic disability in middle-aged populations.

**Findings:**

The MS-VisualPath Cohort was set up to study the neurodegenerative component of MS using advanced imaging techniques by focusing on analysis of the visual pathway in a middle-aged MS population in Barcelona, Spain. We started the recruitment of patients in the early phase of MS in 2010 and it remains permanently open. All patients undergo a complete neurological and ophthalmological examination including measurements of physical and disability (Expanded Disability Status Scale; Multiple Sclerosis Functional Composite and neuropsychological tests), disease activity (relapses) and visual function testing (visual acuity, color vision and visual field). The MS-VisualPath protocol also assesses the presence of anxiety and depressive symptoms (Hospital Anxiety and Depression Scale), general quality of life (SF-36) and visual quality of life (25-Item National Eye Institute Visual Function Questionnaire with the 10-Item Neuro-Ophthalmic Supplement). In addition, the imaging protocol includes both retinal (Optical Coherence Tomography and Wide-Field Fundus Imaging) and brain imaging (Magnetic Resonance Imaging). Finally, multifocal Visual Evoked Potentials are used to perform neurophysiological assessment of the visual pathway.

**Discussion:**

The analysis of the visual pathway with advance imaging and electrophysilogical tools in parallel with clinical information will provide significant and new knowledge regarding neurodegeneration in MS and provide new clinical and imaging biomarkers to help monitor disease progression in these patients.

## Findings

### Background

Multiple Sclerosis (MS) is a chronic immune-mediated disease involving the Central Nervous System (CNS). The prototypical lesions are acute inflammatory plaques, primarily in white matter, that cause demyelination and secondary local axonal damage. These focal lesions are responsible for clinical relapses, the hallmark of the disease [1]. In fact, most patients have an initial relapsing-remitting (RR) course featuring clinical relapses followed by periods of clinical remission. However, after 15–20 years, 65% of patients develop a secondary progressive (SP) phase of the disease characterized by progressive disability independent of relapses [2]. Moreover, some patients experience a primary progressive (PP) form of the disease with areas of inflammation visible on magnetic resonance imaging (MRI) presenting as a gradual accumulation of disability without relapses [3]. As such, relapses cannot completely account for the disability seen in MS. Both histopathological and imaging studies have suggested that diffuse brain damage parallels focal brain damage from MS onset. Furthermore, inflammatory and neurodegenerative mechanisms have been shown to explain diffuse brain damage that involves grey and white matter [4]. Thus, the classical concept stating that focal inflammation leads to secondary axonal damage has been reconceived and recent studies indicate that neurodegeneration occurs independently from inflammation in MS [5]. In fact, neuroaxonal damage is considered to be the main cause of progressive and permanent disability in MS [6]. Immunomodulatory treatments are useful for relapses in the acute inflammatory phase; they prevent disability due to local neuroaxonal damage after relapses but not disability as a consequence of the chronic diffuse neurodegeneration of the disease [3]. For this reason they are effective in the RR phase of MS but not in the SP phase and PP forms [5].

Thus, it is important to consider MS as both an inflammatory and neurodegenerative disease and therefore critical to clarify the mechanisms behind these processes to promote the development of neuroprotective and regenerative therapies. Biomarkers of axonal damage are also needed to assess the efficacy of these new therapies. In the clinical setting, disability is evaluated by Expanded Disability Status Scale (EDSS), which measures neurologic impairment across 8 functional systems. Theoretically, neuroprotective drugs would provide higher benefit in the early phase of the disease because once significant disability is present, corresponding to high EDSS scores (>4.0), it may be too late for protection. Consequently, the aims of this cohort are to evaluate neurodegeneration in MS and to develop clinical and imaging biomarkers of disease severity from the early to the late phases of the disease.

### Rationale: studying neurodegeneration in MS by focusing in the visual pathway

We decided to study neurodegeneration in MS by focusing on the visual pathway for several reasons. First, the visual system is highly susceptible to damage from MS. Optic Neuritis (ON) is a common ophthalmological disorder in these patients [7] and 1 out of 3 MS patients suffer from visual impairment [8]. Moreover, the visual pathway can also be targeted by MS despite a history of prior optic neuritis [9]. Second, the visual pathway can be functionally evaluated with Visual Evoked Potentials (VEP) [10] and visual function tests, namely visual acuity (VA), visual field and color vision. Structural damage to the anterior and posterior visual pathway can be evaluated by Optical Coherence Tomography [11–13] (OCT) and by optic nerve and brain MRI [14–17]. Third, some OCT parameters such as Retinal Nerve Fiber Layer thickness have moderate to high correlations with brain atrophy [18, 19]. Brain atrophy is the most accepted subrogate marker of neurodegeneration in MS and is a predictor for subsequent cognitive decline in MS [20]. The moderate correlation between the retinal nerve fiber layer (RNFL thickness) and cognitive function [21] reinforces the role of visual pathway outcomes as biomarkers of MS. Moreover, both axonal (RNFL) and neuronal components, usually estimated by quantifying the thickness of the Ganglion Cell layer and Inner plexiform layer (GCIP) due to technical limitations to separate these two layers, can be used to monitor neurodegeneration since each showed appropriate correlations with cortical grey matter (p = 0.01 and p = 0.04, respectively) and caudate (p = 0.04 and p = 0.03, respectively) volumes in the eyes of patients with MS without a history of optic neuritis [13]. More recently, Saidha and colleagues presented moderate correlations between GCIP loss and whole brain atrophy (r: 0.44), atrophy in grey matter (r = 0.37), white matter (r = 0.28), and thalamic (r = 0.38) regions of the brain over a 4-year follow-up study [22].

### The aim and objectives

We aim to study neurodegeneration in MS because it determines prognosis and patients’ daily activity. We focus on the afferent visual pathway since it is frequently affected in MS and easily measurable both functionally and structurally. The MS-VisualPath cohort has two main purposes. The first objective is to evaluate the diffuse axonal damage in the early phase of RR-MS form. We aim to assess the role and interplay of different mechanisms including diffuse demyelination, neuroinflammation and axonal degeneration due to acute inflammation, and diffuse trans-synaptic neuroaxonal damage development. The second objective is to identify new biomarkers of neurodegeneration, including brain and retina image biomarkers, as well as more robust clinical markers of disability. Since we began including mfVEP in February 2013, a limited number of participants have baseline values available. For this reason, the role of mfVEP as a biomarker will be evaluated as an exploratory objective.

### Methods

#### The MS-VisualPath cohort

The MS-VisualPath cohort is an ongoing prospective cohort study of patients with MS conducted at the Hospital Clinic of Barcelona, Spain. This hospital provides healthcare to nearly 300,000 habitants in Barcelona. Patients with MS who are followed-up in the center are invited if they fulfil the study criteria. However, patients who are referred to the neurologist of this center can also be included if they show interest in participating. This is an ongoing prospective cohort that started in December 2010 and has permanently open recruitment.

The cohort includes preferentially men or women (age 18–55 years) with RRMS or Clinically Isolated Syndrome (CIS) accordingly to McDonalds criteria [23, 24] with or without history of prior ON. Although the primary objective was to evaluate neuroaxonal damage in early MS (CIS and RR), a group of patients with primary (PP) and secondary (SP) progressive forms were recruited to evaluate differences. Patients need to fulfil the minimum reliability criteria for OCT and visual field. The reliability criteria for OCT includes a well-centered optic disc and fovea, well defined RNFL and Retinal Pigment Epithelium, no artifacts, proper illumination of the fundus, signal strength >20 and no errors in the RNFL and macular automatic segmentation algorithms, thereby fulfilling OSCAR-IB [(O) = obvious problems including violation of the protocol; (S) poor signal strength defined as <15 dB; (C) wrong centration of scan; (A) algorithm failure; (R) retinal pathology other than MS related; (I) illumination; and (B) beam placement] criteria for OCT acquisition [25]. The criteria for visual field are a proportion of false positives and false negatives lower than 20% and 33%, respectively, and fixation losses lower than 20%.

Exclusion criteria are: 1) any progressive neurological disorder (other than MS), any medical condition or limiting psychiatric disease (including depression, bipolar and psychosis) that may interfere with the subject’s ability to cooperate and comply with study procedures; 2) any state of immunosuppression different from MS; 3) any ophthalmological causes for retinal damage different from MS or major difficulties for OCT evaluation such as severe refractive defects [myopia > −6.0 dp or axial eye length >26 mm; hypermetropia > 5 dp; cylinder > 3 dp], optic disk drusen, cataracts, glaucoma; 4) current or previous treatment with a drug involved in toxic neuropathy, such as ethambutol, isoniazid, linezolid, gentamycin, chloramphenicol, vincristine, penicilamine; 5) recent history of acute optic neuritis (<6 months). Patients with ON can be recruited after 6 months of the acute episode; 6) previous diagnosis of Diabetes Mellitus or impaired fasting glucose (≥126 mg/dL or ≥ 200 mg/dL after oral glucose tolerance test); 7) inability to undergo MRI: reduced renal clearance (screening: GFR < 45 ml/min), history of severe hypersensitivity to gadolinium-DTPA, claustrophobia and 8) history of substance abuse in the last 5 years including alcoholism (>40 g/day for women and 60 g/day for men) and severe tobacco use (>20 cigarettes/day). Patients with acute relapses or systemic steroid treatment in the previous month can be included 2 months after the acute episode.

**Figure 1:**
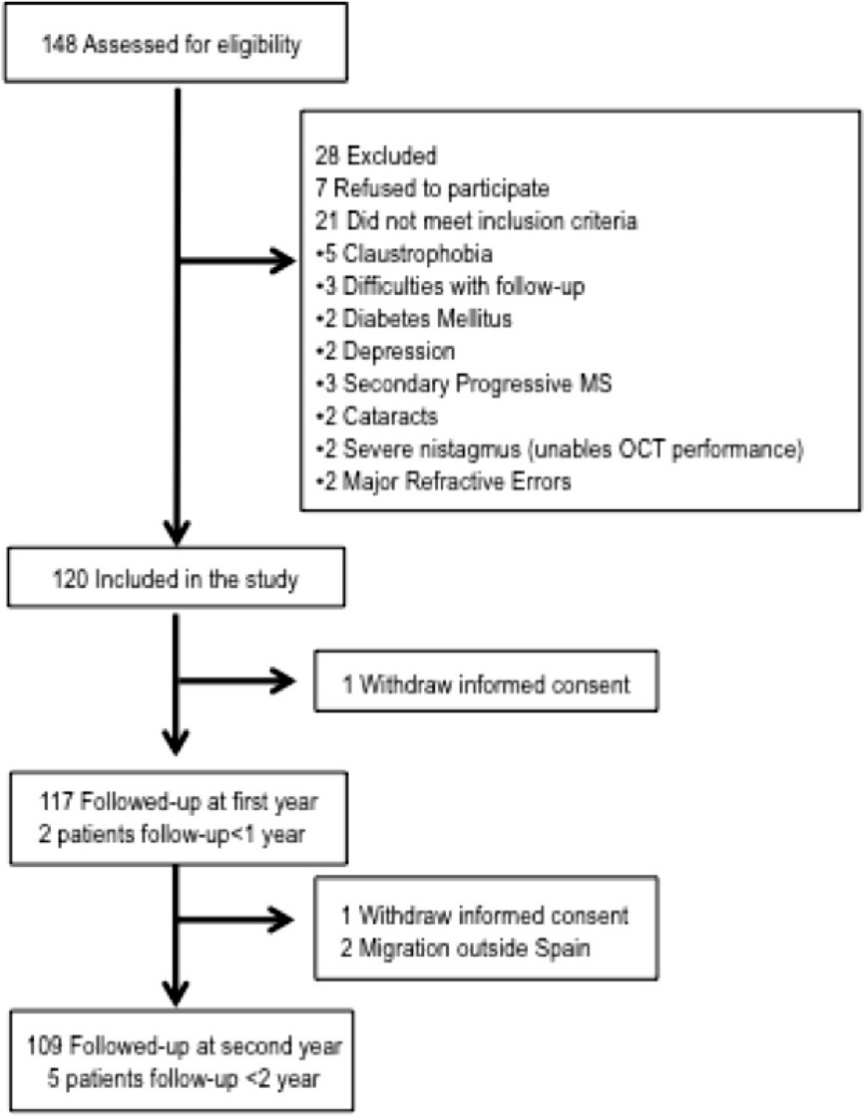
**Displays the flow-chart of participants.** It shows the participants dropped during the recruitment process as well as those dropped during the follow-up.

The selection process begins by extracting names of potential participants from the electronic medical records (EMR) of the Hospital Clinic. The EMR are checked to corroborate that they meet study eligibility criteria. Candidates are approached during their clinical visits and if they show interest in enrolling in the cohort, an interview with the researcher is scheduled. The researcher explains the purpose, visits, and diagnostic tools included in the protocol. If the patient agrees to participate, he/she signs an informed consent and a screening ophthalmological visit is scheduled to evaluate if the participant fulfils the reliability criteria for OCT and visual fields. Most MS patients approached in this way agreed to enrol in the study (Figure 1). Baseline features of the participants are displayed in Table 1.

**Table 1:** **Baseline features of patients included in the MS-VisualPath cohort study**

Demographic and clinical profile (N: 120)	
Sex female n [%]	84 (70)
Age (years)	41.1 ± 9.4
Education >8 years n [%]	107 (89)
Disease duration years	8.6 ± 7.3
Disease Type	
CIS n [%]	7 (6)
RRMS n [%]	103 (86)
SPMS n [%]	6 (5)
PPMS n [%]	4 (3)
Treatment at baseline: yes n [%]	90 (75)
Prior optic neuritis n [%]	52 (43.3)
EDSS	1.8 ± 1.2
ARR 2-year prior recruitment	0.4 ± 0.4

CIS: Clinically Isolated Syndrome; RRMS: Relapsing-Remitting MS; SPMS: Secondary Progressive MS; PPMS: Primary Progressive MS; EDSS: Expanded Disability Status Scale; ARR: Annualized Relapse Rate.

#### Ethics

The Research Ethics Committee approved the study protocol on April 7th, 2009 (Reference: 4905/2009). Data will be kept in accordance with the Spanish Data Protection Law 15/1999 to protect patient confidentially.

#### Data collection

##### Neurological assessment

At baseline, we administer a survey including a set of relevant demographic and clinical variables: sex, date of birth, education level, profession, date of MS onset, date of MS diagnosis, disease subtype, history of prior ON, number of relapses in the prior 2 years before inclusion and disease-modifying therapies for MS.

Disease activity is clinically measured by an annualized rate of relapses and disease severity by EDSS [26] and Multiple Sclerosis Functional Composite (MSFC) [27]. EDSS measures neurological impairment within 8 Functional Systems (pyramidal, cerebellar, brainstem, sensory, bowel & bladder, visual, cerebral, and other) to provide an overall EDSS ranging from 0 (normal) to 10 (death due to MS) [26]. On the contrary, MSFC measures neurological disability and focuses on three major clinical dimensions of MS, namely arm/hand function, leg function/ambulation and cognitive function. MSFC includes the timed 25-foot walk (T25FW) to evaluate lower extremity function, the timed nine-hole peg test (9HPT) to measure upper extremity function, and the 3-second version of the paced auditory serial addition test-3 (PASAT) to assess neuropsychological function [27]. The PASAT test evaluates speed of information processing (executive function), which is the most common and early sign of MS-related cognitive impairment [28]. As the MS Task Force recommends, each raw subscore is converted to a Z-subscore by normalizing to a reference population and then averaged to provide a normalized overall MSFC score [27]. Two-trained neurologists carry out the neurological examinations of participants. We also collect the annualized rate of relapse to clinically measure the disease activity.

In the MS-VisualPath cohort, we estimate cognitive impairment using the Brief Repeatable Battery-Neuropsychology (BRB-N) [29], which includes cognitive tasks to evaluate immediate and delayed verbal memory (Selective Reminder Test - SRT), immediate and delayed visual memory (10/36 Spatial Recall - SPART), executive function (Paced Auditory Serial Addition Test - PASAT - and the Symbol Digit Modality Test - SDMT) and verbal fluency (Word List Generation - WLG), using Spanish normative cut-offs [30]. Two neurologists administer BRB-N after receiving additional training by an MS neurologist experienced in the cognitive evaluation of MS using neuropsychological batteries.

##### Visual function testing

We assess high contrast (HCVA) and low contrast visual acuity (LCVA). Since ON attacks (even subclinical episodes) are frequent in MS, we prefer to evaluate monocular VA. Moreover, this approach facilitates assessment of functional-structural correlations with OCT and VEP. After correcting for any refractive errors, including in patients with eyeglass prescriptions, we assess HCVA and LCVA with Early Treatment for Diabetic Retinopathy Study (ETDRS-Precision Vision, LaSalle, IL) visual acuity charts and new Low-Contrast Sloan Letter Charts (LCSLC-Precision Vision, LaSalle, IL), respectively. ETDRS and LCSLC charts are printed on a 14 ½ × 13 1/2–inch durable laminated board. ETDRS charts have black letters of progressively smaller size on a white backboard, and each line represents a different VA. LCSLC have grey letters of progressively lower contrast on a white backboard and each line represents a certain amount of contrast sensitivity. We selected 2.5% and 1.25% contrast levels as representative values of LCVA. These levels of contrast have been successfully used in MS to assess visual function [31–33]. A lighting level of 80–100 foot-candles measured at the white non-letter portions of the charts is maintained during the visual acuity evaluation as has been previously published [31–33]. A trained optometrist asks participants to read the charts from a distance of 4 meters. First, the optometrist evaluates monocular high visual acuity for each eye and after that, low visual acuity independently for each eye. The number of correctly identified letters is recorded for each eye of each participant. Scores range from 0 to 70 letters. Visual acuity is also scored as a Logarithm of the Minimum Angle of Resolution (logMAR). The formula is VAlogMAR = 0.1 + LogMAR value of the best line read - 0.02 X (number of letters). Since some authors suggested that binocular vision may better reflect visual disability [34], we have added binocular 2.5% LCVA in the third year evaluation.

Color vision is tested using Hardy, Rand and Rittler (HRR) pseudoisochromatic plates. The HRR test has 24 plates with 0, 1 or 2 symbols depending of the plate. There are three types of plates: 4 non-scored demonstration plates; 6 scored screening plates and 14 scored plates for type and severity assessment. The first four plates (plates 1–4) are used to ensure understanding and enough visual acuity to perform the task. The 6 screening plates (plates 5–6 with 4 symbols for blue-yellow deficit and plates 7–10 with 6 symbols for red-green) classify eyes as having dyschromatopsia or normal color vision. An eye with less than two errors is considered normal. There is no need to continue the test if the HRR score is 35 (one error) or 36 (zero errors). We used two errors as a cut-off point to ensure a sensitivity of 1.0, as has been previously described [35]. An eye with two or more errors in the screening plates has impaired color vision and plates 11–24 should be administered to determine type and extent of the defect. Plates 11–20 (18 symbols) and plates 7–10 are used to assess red-green defect with three levels of severities: mild (plates 7–10 with 6 symbols and plates 11–15 with 8 symbols); moderate (plates 16–18; 6 symbols) and severe (plates 19–20, 4 symbols). Plates 21–24 (8 symbols) and plates 5–6 are used to evaluate blue/yellow defect with three levels of severities: mild (plates 5–6); moderate (plates 21–22; 4 symbols) and severe (plates 23–24; 4 symbols). The last group of plates in which errors occur establishes the extent of the color deficit. For instance, if the last error occurs in either plates 7–10 or 11–15 and there are no errors in plates 16–20 there is a mild red-green deficit. In our study, color vision is evaluated qualitatively based on the number of errors in the screening plates: an eye has a color vision deficit if it has at least two errors in the screening plates. Moreover, color vision was measured quantitatively based on the number of correctly identified symbols in the 20 scored HRR plates with a maximum of 36 symbols.

Visual fields are evaluated with Humphrey Field Analyzer 750 (Carl Zeiss Meditec, Inc) using Swedish Interactive Threshold Algorithm (SITA) - standard central 24–2 protocol. The stimulus is a Goldman size III (0.43 Deg) with a background luminance of 31.5 Apostlibs. We defined the reliability criteria for this test in the previous section. Any visual field not matching these criteria is repeated or excluded after two failed repetitions. An abnormal visual field is defined by a mean deviation of the total deviation plot with p < 0.05.

##### Quality of life assessment

We evaluate the quality of life of MS patients using the SF-36v2™ scale. This health survey is a multi-purpose short-form scale that includes physical, mental and social functional health measures [34]. We also assess the presence of anxiety and depressive symptoms in the cohort, even though we excluded participants with severe mood disorders. We use the Hospital Anxiety and Depression Scale (HADS). This scale, originally proposed by Zigmond AS and Snaith RP for detecting depression and anxiety in the setting of hospital medical outpatient clinic [36], has been successfully used for evaluating MS patients [37]. HADS has been validated in the Spanish population [38].

Finally, we use the 25-Item National Eye Institute Visual Function Questionnaire (NEI-VFQ-25) and the 10-Item Neuro-Ophthalmic Supplement to specifically assess visual disability. NEI-VFQ-25 includes questions representing 11 vision-related issues [general vision, ocular pain, difficulty with near activities, limitations in distance activities, vision specific limitations in social functioning due to vision, role limitations due to vision, dependency on others due to vision, mental health symptoms due to vision, driving difficulties, color vision and peripheral vision limitations] and a single-item general health-rating question. Scores of NEI-VFQ-25 range from 0 to 100 with higher scores indicating better visual functioning [39]. The 10-Item Neuro-Ophthalmic Supplement was designed to target additional aspects of visual disability among patients with neuro-ophthalmological conditions such as ON. Scores also range from 0 to 100 [40]. Both questionnaires have been previously used in MS [41, 42]. All health-related quality of life surveys are suitable for self-reported administration.

##### Visual pathway electrophysiological assessment

Multifocal Visual Evoked Potentials (mfVEP) are recorded using the monocular VisionSearch1 perimetry system (Vision Search, Sydney, Australia). The visual stimulus is generated on a high-resolution LCD display and the patient should be comfortably seated 35 cm from the screen. The stimulus is cortically scaled with an eccentricity dartboard pattern comprised of 56 segments. Each segment contains a 4×4 checkerboard pattern of black and white squares. The size of each square is proportional to the size of the segment and dependent on eccentricity. Stimulus conditions require luminance of 146 candela (cd) per m^2^ for white squares and 1.1 cd/m^2^ for black squares with a contrast of 99%. Two opposite binary reversal sequences occur at each of the 56 sites in the visual field according to a pseudorandom sequence. Each stimulation site is modulated in time according to a different sequence. The final signal is computed by cross-correlation of the response evoked by the sequence stimulation with the sequence itself for each site or area of visual field. To record, we use four gold-cup electrodes (Grass Technologies, West Warwick, RI, USA) placed on the scalp (2 electrodes either side of the inion, one electrode 2.5 cm above and one 4.5 cm below the inion in the midline). Electrical signals are recorded along 2 channels (differences between superior and inferior and left and right electrodes). MfVEP are amplified 1 × 10^5^ times and band-pass filtered 1 Hz to 20 Hz using a Grass 15LT amplifier. The recordings are collected using monocular stimulation and repeated 6–10 times in order to reduce the signal/noise ratio. Then, correlations between pattern reversal sequences and recorded electrical signals are performed with Terra™ software (Vision Search, Sydney, Australia) to obtain responses for each segment.

The largest peak-trough amplitude within the 70–210 msec is determined for each channel. For amplitude analysis, the software automatically designates the wave of maximal amplitude between two channels and a combined topographic map is created. For latency analysis, the second peak of the largest wave from two channels of a given visual field segment of both eyes is automatically selected by a specially designed algorithm. The same channel and same peak are then used to assess latency for a particular segment in the other eye. Ultimately, mfVEP determines inter-eye differences in the latency and amplitude of the cortical response in each segment. It is also possible to compare latencies and amplitudes of a patient’s eye and normative data of a healthy population.

This method for mfVEP performance has shown good technical feasibility and consistency in a study including healthy controls and ON patients from two different countries [43]. Latencies of mfVEP may be useful to discriminate patients with ON at risk of MS progression [44]. Moreover, some authors have found a strong correlation between latency delay of mfVEP and axonal damage measured by RNFL [10] and mfVEP amplitude and disability measured by EDSS [45].

#### Image assessment

##### Wide-field fundus imaging

A trained ophthalmologist performs a complete indirect ophthalmoscopy after full mydriasis at baseline. For follow-up assessment, we perform wide-field fundus imaging using Optomap200 (Optos®). This optical device consists of a scanning laser ophthalmoscope that provides images of a 200° field of the retina without mydriasis. This system images 80% of the retina in a single shot [46]. Optomap200 has a moderate sensitivity for lesions located posterior to the equator but low sensitivity for those situated anterior to the equator [47]. This limitation is explained by the low image quality in the far temporal and nasal peripheral retina and the fact that the peripheral superior and inferior peripheral retina cannot be scanned [48].

##### Optical coherence tomography

Optical Coherence Tomography is performed for each eye by a trained optometrist using two OCT systems: the Blue Peak™ Spectral Domain OCT Spectralis® (Heidelberg Engineering, Germany) and the 4000 Spectral Domain OCT Cirrus® (Carl Zeiss, Dublin, US). The protocol for Spectralis was as follows: (1) RNFL Thickness: raster scan centered on the optic nerve head (ART = 100 frames; diameter 12°; which equals approximately a 3.5 mm diameter ring in a normal sized eye) and (2) Macular Volume: raster scan centered on the fovea (ART > 9frames; matrix size 20 × 20; 25 sections of 240um; 6 mm ring area; horizontal acquisition). The protocol for Cirrus was as follows: an optic disc cube 200 × 200 centered on the disk was used to measure RNFL thickness and a macular cube 512 × 128 centered on the fovea was used to assess macular volume (MV) both with signal strength > 5/10. Both optical devices have demonstrated adequate reproducibility of OCT [49, 50] measures although Spectralis equipment provides TruTrack™ active eye tracking technology to further improve reproducibility of measurements.

To be included in the study all OCT needs to fulfil OSCAR-IB criteria: [(O) = obvious problems including violation of the protocol; (S) poor signal strength defined as <15 dB; (C) wrong centration of scan; (A) algorithm failure; (R) retinal pathology other than MS related; (I) illumination; and (B) beam placement] [25]. A trained optometrist performs OCT and assesses whether each fulfils the OSCAR-IB criteria in our cohort. In cases where OCT does not fulfil the criteria, acquisition is repeated or the data is ultimately excluded. Our criteria for exclusion includes any ophthalmological causes for retinal damage other than MS (R) as well as any major difficulties in OCT evaluation including cataracts and other opacities leading to poor signal strength (O and S). Most of our patients have mild retinal atrophy in which algorithm failures are less probable (A). In our cohort, most rejections are due to patients moving their heads, resulting in artifacts on peripapilar scans and missing slices on macular volume scans.

##### Brain magnetic resonance imaging

Brain images are acquired using a Siemens Trim Trio® 3 Tesla with a 32-channel phased array coil. The following sequences are obtained at baseline: a) 3D structural T1-MPRAGE (Magnetization-Prepared Rapid-Gradient-Echo) [voxel size 0.9 × 0.9 × 0.9 mm^3^, Field of View (FOV) 220 mm, Flip angle 9°, Repetition Time (TR): 1970 ms, Echo Time (TE): 2.4 ms, Inversion recovery time (TI): 1050 ms]; b) 3D Structural FLAIR (Fluid Attenuated Inversion Recovery) [Voxel size 0,9 × 0,9 × 0,9 mm^3^, FOV 220 mm, TR: 5000 ms, TE: 393 ms TI: 1800 ms]; c) 2D Axial T1 post gadolinium [Voxel Size: 0.7 × 0.6 × 3.0 mm^3^; FOV 240 mm, TR: 390 ms, TE: 2.65 ms]; d) Single voxel proton-spectroscopy (H^1^MRS) [TR 3000 ms, TE 144 ms, volume of interest (VOI) size 20 × 30 × 20 mm^3^, including In-vivo water suppressed (128 averages) and water unsuppressed (16 averages) acquisitions]. The H^1^MRS VOI was positioned in the visual cortex (inter-hemispheric fissure, including both calcarine sulci) and in the precentral cortex. The absolute N-Acetyl Aspartate [NAA] in the visual cortex was calculated using LCModel software 7; e) Diffusion Tensor Imaging (DTI) [Voxel Size 2.5 × 2.5 × 2.5 mm^3^; FOV: 240 mm; b-val 1000 s/ mm^2^, NG: 30, Nb0: 1, TR: 6900; TE: 89] f) Coronal T2 fat saturated spin echo acquisitions for each optic nerve [Voxel Size 0.5 × 0.5 × 2 mm^3^, 20 parallel slices transversal to optic nerve, flip angle 120°, TR: 2600 ms and TE: 83 ms] and Coronal T1 for each optic nerve [Voxel Size 0.5 × 0.5 × 2 mm^3^, 20 parallel slices transversal to optic nerve, flip angle 70°, TR: 483 ms and TE: 4.92 ms] before and after gadolinium. These sequences are acquired before GD injection.

During the follow-up, we have made some changes: a) exclusion of the optic nerve sequences: b) substituting HARDI (High-Angular Resolution Diffusion Imaging) in place of DTI scans [2 × 2 × 2 mm^3^; FOV: 240 mm; b-val 1500 s/mm^2^, NG: 70, Nb0:6; TR: 12600 ms; TE:112 ms] because we have found a better track reconstruction with HARDI (manuscript in preparation) c) adding Resting State functional MRI (RS-fMRI) [Voxel Size 1.7 × 1.7 × 3 mm^3^; TR: 2000 ms; TE: 19 ms; Slices: 40; Flip angle 90]. The only sequences acquired after gadolinium injection are 2D Axial T1 post gadolinium and optic nerve sequences in the old protocol, and 2D Axial T1 post gadolinium in the new one.

#### Follow-up assessment

Patients are examined yearly for 3 years, but 5-years follow-up and thereafter are already considered. After the third follow-up, patients will be asked to enrol in the 2-year extension study. Table 2 shows the data collected in each follow-up visit in the MS-VisualPath cohort. Figure 1 shows flow-chart of participants in the MS-VisualPath cohort. The attrition rate after 2 years of follow-up is 3.3%. In fact, half of these participants dropped out because they moved to another region of Spain (one patient) or another country (one patient).

**Table 2:** **Data assessment in the MS-VisualPath Cohort, Barcelona, Spain**

	Baseline	Year 1	Year 2	Year 3	Year 4	Year 5
EDSS and Annualized Relapse Rate	X	X	X	X	X	X
Multiple Sclerosis Functional Composite	X	X	X	X		X
Quality of Life (SF-36, NEI-VFQ-25 + 10S^1^)	X			X		X
Anxiety and Depression assessment^1^	X			X		X
Neuropsychological Battery (BRB-N)	X		X			X
High (EDTRS) and Low (LCSLC) VA	X	X	X	X	X	X
Color vision test (HRR plates)	X	X	X	X	X	X
Visual Fields	X	X				X
Indirect Funduscopy - Optomap200^2^	X	X	X	X	X	X
Multifocal Visual Evoked Potentials^2^	X	X	X	X	X	X
Optical Coherence Tomography	X	X	X	X	X	X
Magnetic Resonance Imaging	X	X	X	X	X	X

Included in the protocol on ^1^ September 2013 and ^2^February, 2013. EDSS: Expanded Disability Status Scale; MSFC: SF-36: Short Form-36; NEI-VFQ-25 + 10S: 25-Item National Eye Institute Visual Function Questionnaire (NEI-VFQ-25) and the 10-Item Neuro-Ophthalmic Supplement; BRB-N: Brief Repeatable Battery-Neuropsychology; EDTRS: Early Treatment for Diabetic Retinopathy Study; LCSLC: Low-Contrast Sloan Letter Chart; VA: Visual Acuity; HRR: Hardy Rand and Rittler.

### Statistics

This study collects a wide range of results that will be examined using the following standard approach. First, we will perform descriptive statistics to characterize the sample using absolute numbers and proportions for qualitative variables and mean and standard deviation for quantitative variables. Data will be tested for normality, homoscedasticity and independence to test assumptions used for parametric tests. Then, we will perform bivariate analyses using a X^2^ test or Fisher test (in case of small samples) for qualitative variables and independent 2-sample t-test or ANOVA (three or more groups) for quantitative variables. For data that is not parametric, we will use the U-Mann–Whitney test. Pearson’s Correlation test or the non-parametric analogue, Spearman rank order correlation coefficient, will be used to evaluate correlations. Finally, we will perform multivariate tests to rule out confusion that is inherent to cohort studies. Two-tailed p-values <0.05 will be considered statistically significant. All analyses will be performed with the Statistical Package IBM-SPSS (SPSS Inc, Chicago, IL, USA) software version 20.0 or superior.

### Findings

#### Transynaptic degeneration in MS patients: evidence from the MS-VisualPath cohort

We have evaluated transynaptic degeneration in a cross-sectional and short-term follow-up study including the first 100 consecutive MS patients in the MS-VisualPath. We found that visual cortex volume, NAA in the visual cortex, and lesion volume within optic radiations significantly influenced average RNFL thinning independently of other confounders, especially optic neuritis. Additionally, patients with severe prior ON had lower visual cortex volume than patients without ON [51].

#### Retinal periphlebitis is associated with disease severity

Considering our focus on development of biomarkers of axonal damage, we have found that patients with previous retinal periphlebitis had a tendency towards a higher disability at baseline and disability progression after a 1-year follow up compared to patients without primary retinal inflammation. Specifically, these patients showed higher lesion volume, lower brain volume and lower RNFL thickness [all of which are surrogate markers of axonal damage] than patients without primary retinal inflammation [52]. We evaluated the association between periphlebitis and clinical and paraclinical markers of neurodegeneration, including RNFL thickness, using regression analyses. We ran general linear models adjusted for sex, age, disease duration, and use of MS treatment considering RP as an independent (predictor) variable and RNFL, MV, normalized brain parenchymal volume (and GM / WM volumes), lesion volume and EDSS as dependent (predicted) variables in separate models. Since the outcome variables are quantitative, we cannot estimate true positive and true negative variables and as such, accuracy (TP + TN/overall) cannot be calculated.

#### Color vision impairment is associated with disease severity

In addition, we explored the potential role of color vision function testing as a marker of disability and neurodegeneration. We have found that patients with dyschromatopsia in non-ON eyes had greater disability and axonal damage than MS patients with normal color vision at baseline. Moreover, patients who developed incident dyschromatopsia after 1-year follow-up displayed more disability progression and axonal damage than those without dyschromatopsia [53]. We used the same statistical approach that we performed for retinal periphlebitis to evaluate color vision.

### Discussion

It is our belief that the results of the MS-VisualPath Cohort study will provide new and significant knowledge regarding neurodegeneration in MS as well as new clinical and imaging biomarkers to help monitor disease progression in these patients. However, we acknowledge some limitations of our study. In the MS-VisualPath cohort, all the hypotheses and studies should take into account the effect of prior ON on visual function testing, OCT and MRI values. The presence of prior history is assessed by patient self-report and confirmed after reviewing the EHR as described in the Optic Neuritis Treatment Trial [54]. However, subclinical ON is difficult to rule out. In the MS-VisualPath Cohort, we consider optic nerve MRI and OCT acquisitions and visual field criteria to evaluate subclinical ON. A subject is considered to have subclinical unilateral ON if all of the following findings are detected: the presence of unilateral focal signal hyperintensity of optic nerves in two or more contiguous MRI slices and the presence of abnormal inter-ocular asymmetry both in RNFL thickness and in mean deviation of visual field. We considered these asymmetries as abnormal if RNFL thickness or the visual fields mean deviation were above the mean plus one standard deviation of inter-ocular asymmetry of cases with self-reported history of prior ON. The MS-VisualPath is an open prospective study so new candidates will be also screened by mfVEP, which are very sensitive in detecting subclinical ON [55]. Furthermore, our MRI protocol does not include any sequences with improved sensitivity to detect cortical lesions that may contribute to axonal damage in the early RRMS, such as Double Inversion Recovery [56] or Phase Sensitive Inversion Recovery [57]. Nevertheless, our study also has strengths. In the MS-VisualPath cohort, the neurologic and ophthalmologic examinations are performed with validated tools and performed by trained specialists on a multidisciplinary team (optometrist, ophthalmologist and neurologist). Another major strength of the MS-VisualPath cohort is that the retention rate after 2 years of follow-up is nearly 97%. A high retention rate is a major methodological requirement in prospective studies. Finally, these MS patients are frequently followed-up with in the neurology outpatient consultancy, so the close monitoring of participants is also strength of the MS-VisualPath cohort.

### Conclusion

This MS-VisualPath study has provided the opportunity to study the pathological mechanisms leading to neurodegeneration and disability. We have also provided two easily assessed clinical biomarkers, namely periphlebitis and dyschromatopsia, related to neurodegeneration. Future analyses and results will further extend these objectives. However, we acknowledge that our results should be externally validated in other cohorts. Thus, collaborations with national and international studies are welcome and can be proposed to: pvilloslada@clinic.ub.es. Scientific proposals must be satisfactorily peer-reviewed and ethically reviewed and approved. Contact details, publications and data request are available upon request (http://www.neuroimmunologybcn.org).

## Abbreviations

9HPT: 9 Hole Peg Test
BRB-N: Brief repeatable battery-neuropsychology
CIS: Clinically isolated syndrome
CNS: Central nervous system
DTI: Diffusion tensor imaging
EDSS: Expanded disability status scale
EHR: Electronic health records
ETDRS: Early treatment for diabetic retinopathy study
FLAIR: Fluid attenuated inversion recovery
FOV: Field of view
HADS: Hospital anxiety and depression scale
HARDI: High-Angular resolution diffusion imaging
HCVA: High contrast visual acuity
HRR: Hardy, rand and ritter
LCSLC: Low contrast sloan letter charts
LCVA: Low contrast visual acuity
LogMAR: Logarithm of the minimum angle resolution
mfVEP: Multifocal visual evoked potentials
MPRAGE: Magnetization-Prepared Rapid-Gradient-Echo
MRI: Magnetic resonance imaging
MSFC: Multiple sclerosis functional composite
MS: Multiple sclerosis
MV: Macular volume
NAA: N-Acetyl-Aspartate.
NEI-VFQ-25: 25-Item National eye institute visual function questionnaire
OCT: Optical coherence tomography
ON: Optic neuritis
PASAT: Paced auditory serial addition test-3
PP: Primary progressive
RNFL: Retinal nerve fiber layer
RR: Relapsing-remitting
RS-fMRI: Resting state functional magnetic resonance imaging
SDMT: Symbol digit modality test
SITA: Swedish interactive threshold algorithm
SP: Secondary progressive
SPART: 10/36 Spatial Recall
SRT: Selective reminder test
TR: Repetition time
TE: Echo time
TI: Inversion recovery time
T25FW: Time 25-Foot Walk
VA: Visual acuity
VOI: Volume of interest
VEP: Visual evoked potentials
WLG: Word list generation.
